# Therapeutic Targeting of HIV Reservoirs: How to Give T Cells a New Direction

**DOI:** 10.3389/fimmu.2018.02861

**Published:** 2018-12-04

**Authors:** Hongbing Yang, Zoë Wallace, Lucy Dorrell

**Affiliations:** ^1^Nuffield Department of Medicine, University of Oxford, Oxfordshire, United Kingdom; ^2^Immunocore Ltd., Oxon, United Kingdom; ^3^Oxford NIHR Biomedical Research Centre, University of Oxford, Oxford, United Kingdom

**Keywords:** HIV reservoirs, T cells, CAR (chimeric antigen receptor) T cells, dual affinity re-targeting (DART), T cell receptor (TCR), clinical trial, kick and kill

## Abstract

HIV cannot be cured by current antiretroviral therapy (ART) because it persists in a transcriptionally silent form in long-lived CD4+ cells. Leading efforts to develop a functional cure have prioritized latency reversal to expose infected cells to immune surveillance, coupled with enhancement of the natural cytolytic function of immune effectors, or “kick and kill.” The most clinically advanced approach to improving the kill is therapeutic immunization, which aims to augment or re-focus HIV-specific cytolytic T cell responses. However, no vaccine strategy has enabled sustained virological control after ART withdrawal. Novel approaches are needed to overcome the limitations of natural adaptive immune responses, which relate to their specificity, potency, durability, and access to tissue reservoirs. Adoptive T cell therapy to treat HIV infection was first attempted over two decades ago, without success. Since then, progress in the field of cancer immunotherapy, together with recognition of the similarities in tumor microenvironments and HIV reservoirs has reignited interest in the application of T cell therapies to HIV eradication. Advances in engineering of chimeric antigen receptor (CAR)-transduced T cells have led to improved potency, persistence and latterly, resistance to HIV infection. Immune retargeting platforms have incorporated non-neutralizing and broadly neutralizing antibodies to generate Bispecific T cell Engagers (BiTEs) and Dual-Affinity Re-Targeting proteins (DARTs). T cell receptor engineering has enabled the development of the first bispecific Immune-mobilizing monoclonal T Cell receptors Against Viruses (ImmTAV) molecules. Here, we review the potential for these agents to provide a better “kill” and the challenges ahead for clinical development.

## Introduction

Long-lived cells that harbor replication-competent HIV are responsible for viral persistence during antiretroviral therapy (ART). Within the first few days of infection, HIV-1 inserts into its host cell genome, primarily in CD4+ T cells that are transitioning from an activated to a quiescent state (1–4). Other cell populations of lymphoid, myeloid, and stromal origins also contribute to HIV reservoirs; however, the mechanisms involved are less well-understood, in part due to the challenges of sampling tissues (5). The persistence of these reservoirs has been explained by clonal proliferation of T cells and the existence of sanctuary sites, which may be both the cause and consequence of inadequate antiretroviral drug penetration and ineffective adaptive immune responses (6–11). The lack of a unique and reliable marker of these diverse reservoirs has significantly encumbered the development of strategies to provide either a sterilizing cure, in which all forms of HIV are eliminated from the body, or a functional cure (remission), defined by long-term control of HIV and preserved immune competence after ART withdrawal (12, 13). Only one person has achieved a sterilizing cure and cases of sustained antiretroviral-free HIV control remain rare (14, 15). However, advances in quantification and characterization of viral reservoirs, coupled with an unprecedented expansion in cancer immunotherapeutics to counteract immune exhaustion have opened up new possibilities for their application to the eradication of HIV reservoirs (16–18). In addition, it is possible to limit seeding of reservoirs and viral escape by initiating ART during primary infection, which may improve the chance of achieving a functional cure (19–21).

## How Can Immune Retargeting Therapies Overcome the Failure of Natural Immune Responses to Eliminate HIV Reservoir Cells?

Several barriers to clearance of HIV reservoirs by the host immune response need to be considered when assessing the potential benefits and limitations of T cell retargeting agents: (1) low or absent viral antigen expression during ART; (2) viral heterogeneity resulting from mutational escape; (3) T cell dysfunction and/or exhaustion; (4) the inaccessibility of reservoir cells (22–25).

Viral latency is a state of reversible non-productive infection within a cell, which permits immune evasion. The rationale for using latency-reversing agents, the kick in “kick and kill,” is to initiate viral gene transcription and protein synthesis, thus removing protection from immune surveillance (26–28). However, recent developments in characterizing the molecular composition of the reservoir suggest that this may be overly simplistic. The vast majority of latently infected cells harbor defective proviruses, yet they may be transcriptionally active; translation of open reading frames with intact gag and nef sequences can lead to protein synthesis and susceptibility to killing by CD8+ T cells *in vitro* (29–31). Boosting of CD8+ T cells by therapeutic vaccination, with or without latency reversal, has not been successful in reducing viral reservoirs. This may reflect targeting of irrelevant epitopes, persistent T cell dysfunction and limited potency of LRAs (32–35). Furthermore, cells harboring intact and inducible proviruses may be inherently resistant to CD8+ T cell killing (36). Individuals who spontaneously control HIV have smaller latent reservoirs and display functionally superior CD8+ T cell responses, providing a model for functional cure (37, 38). However, loss of controller/non-progressor status is frequent, possibly due to ongoing viral replication in tissue sites that are inaccessible to cytolytic T cells (39–41).

**Figure 1:**
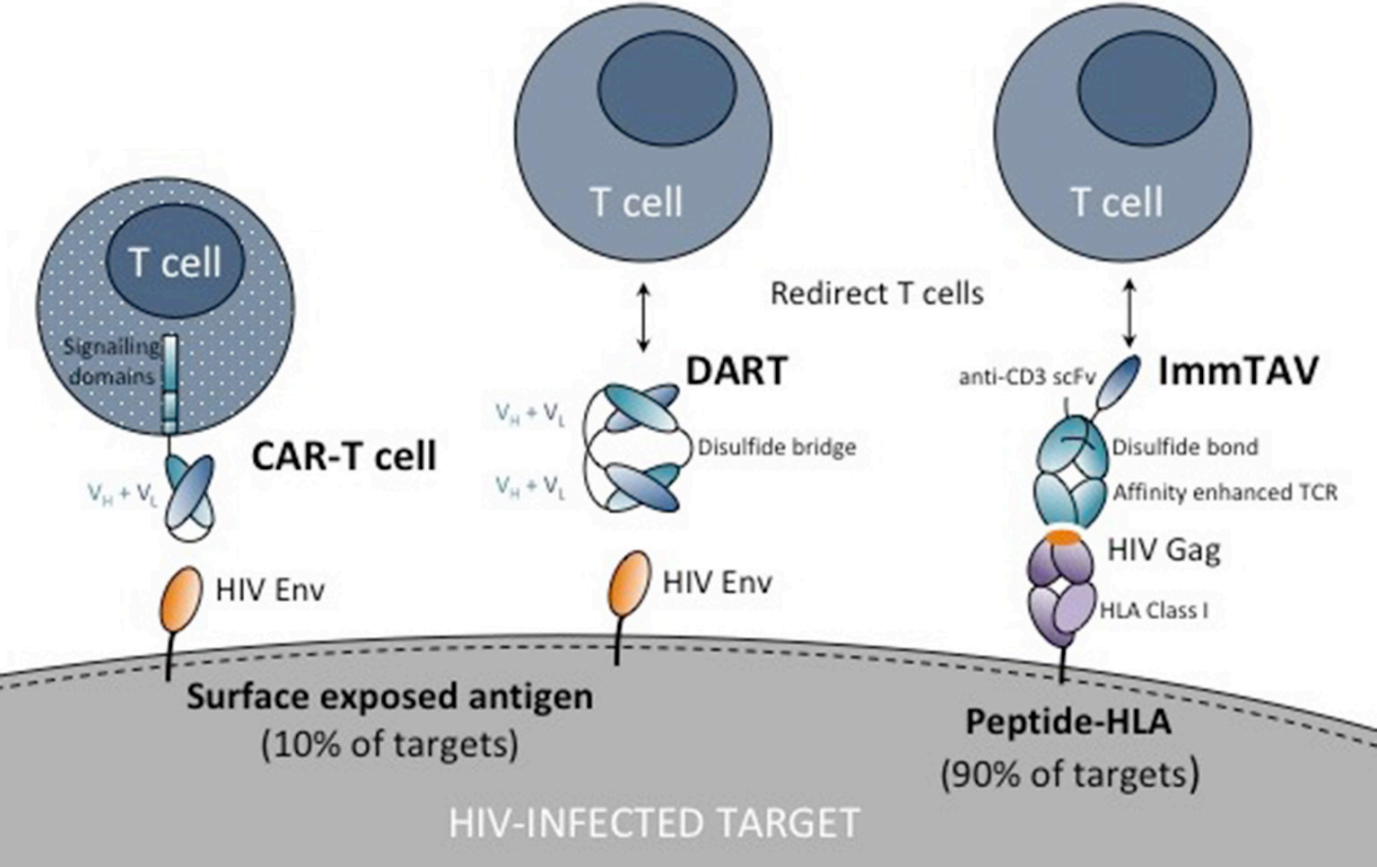
Schematic showing chimeric antigen receptor (CAR) T cell, dual affinity retargeting (DART) and immune-mobilizing monoclonal T cell receptor against viruses (ImmTAV) antigen recognition domains (antibodies or T cell receptors shown as blue ovals) and their respective targets on HIV-infected cells. The CAR is fused to one or more intracellular signaling domains. DARTs and ImmTAVs initiate signaling in T cells through cell surface CD3 via an anti-CD3 single chain variable fragment (scFv) which is fused to the antibody/TCR by a flexible linker (black line).

In this review, we discuss the potential for T cell retargeting therapies to bring about a functional cure by overcoming the hurdles outlined above, namely, overcoming low antigen expression through affinity enhancement of antigen receptors, mobilizing sufficient numbers of effectors targeting conserved or non-escaped viral epitopes, recruiting functionally intact cells, and exploiting technologies to optimize tissue penetration and persistence (Figure 1). In addition, we examine the safety implications and the challenges for delivering these therapies to patients. Although adoptive T cell therapy, with or without TCR gene transfer, was the forerunner of these technologies and new adapted approaches are showing promise, this is beyond the scope of the discussion and is comprehensively covered elsewhere (42, 43).

## Chimeric Antigen Receptor (CAR) T Cells

CAR technology has evolved over more than two decades. It provides a means to re-programme T cells to recognize cell surface proteins through gene transfer of synthetic chimeric antigen receptors (CAR) (monoclonal antibodies) fused to a T cell activation domain. While the repertoire of potential CAR targets is smaller than that of T cell receptors, antigen recognition is not HLA-restricted, which is an advantage over conventional adoptive T cell therapy. Furthermore, CARs exploit healthy T cells that do not display the immune exhaustion phenotype typical of HIV-specific T cells in chronic infection.

The first anti-HIV CAR comprised the extracellular region of CD4 fused to a CD3ζ signaling domain (CD4ζ-CAR), conferring specificity for HIV-infected cells through binding of CD4 to the envelope protein, gp120. However, despite evidence of antiviral efficacy *in vitro*, CD4ζ-CAR T cells infusions had minimal impact on plasma viraemia in ART-naïve subjects or on viral reservoirs in ART-suppressed patients (44, 45). Modest cytolytic function *in vivo*, limited persistence due to poor proliferative capacity and susceptibility to HIV infection may have contributed to these disappointing results. Improvements in the technology were achieved through successive modifications in the signaling domains to enhance anti-tumor activity: second- and third-generation CARs were produced by addition of one or more costimulatory domains such as 4-1BB, CD28 and ICOS (46–48). Second-generation CD19-specific CARs achieved spectacular response rates in the treatment of B cell malignancies (albeit with high levels of toxicity) leading to their approval by the US Food and Drug Administration and paving the way for the development of more effective HIV-specific CARs (49–51). A re-engineered CD4 CAR containing the 4-1BB domain showed significantly enhanced antiviral potency *in vitro* and greater capacity to proliferate and prevent HIV spread in a humanized mouse model than the first-generation version (52). In addition, a large number of broadly neutralizing antibodies (bNAbs), which target regions of vulnerability in the viral envelope, have since been identified as potential CAR candidates (53).

Achieving sustained virological control after ART cessation will likely require repeated infusions of CAR T cells or strategies to prolong their persistence *in vivo*. One approach is to engineer the expression of CD4ζ CAR in hematopoietic stem cells (HSC). Modified HSC-derived CAR T cells were shown to engraft, proliferate, and display antiviral activity in a humanized mouse model of HIV infection; however, in a non-human primate model only transient and partial control of viral replication was observed after SHIV challenge and withdrawal of ART (54, 55).

A further consideration is to protect modified T cells from HIV infection. The safety and efficacy of zinc finger nuclease disruption of CCR5, the co-receptor for viral entry into CD4+ T cells has been demonstrated in a clinical trial: modified autologous CD4+ T cells had a mean half-life of 48 weeks during ART and were maintained for longer than unmodified CD4+ T cells after ART withdrawal (56). To generate functional HIV-resistant CAR T cells, site-directed megaTAL nuclease-mediated CCR5 gene disruption has been successfully combined with targeted delivery of an adeno-associated virus CAR cassettes to the CCR locus using homology-directed recombination/repair (HDR), which achieves highly efficient integration (57). Simultaneous CCR5 gene disruption and expression of single chain variable fragments (scFv) of HIV-specific broadly neutralizing antibodies (bNAb) coupled to a second-generation co-stimulatory domain in primary human T cells endowed them with the capacity to suppress viral replication *in vitro* (57, 58). However, feasibility of manufacture for clinical application has yet to be demonstrated.

B cell follicles in lymphoid tissue are a sanctuary site for HIV replication, which occurs preferentially in T follicular helper cells, due to exclusion of CD8+ T cells that lack expression of the follicular homing chemokine receptor CXCR5 (59). Haran et al. successfully co-expressed CXCR5 with an antiviral bispecific CAR comprising rhesus CD4 domains fused to mannose-binding lectin, which targets carbohydrate residues on the viral envelope. SIV CAR/CXCR5-expressing T cells were able to suppress SIV replication *in vitro* and to migrate to B cell follicles in lymphoid tissue explants (60).

Two clinical trials of CAR T cell therapy in HIV-positive ART-treated subjects are ongoing or due to recruit: one is evaluating a bNAb (VRC01)-based CAR, VC-CAR-T (NCT03240328) and the other, a CD4 CAR modified for HIV resistance by ZFN disruption of CCR5 and conjugation of the C34 peptide to the CXCR4 N-terminus (NCT03617198) (61).

## Bispecific Antibodies

Bispecific antibodies (bsAb) have emerged as a more scalable alternative to CAR T cell therapy. Originally developed to overcome the limitations of monoclonal antibodies for cancer therapy, the first generation bsAb platform, bispecific T cell engagers (BiTEs), comprised a tumor-specific variable fragment and a CD3-specific fragment joined by a single peptide linker. This format enabled redirection of effector T cells to eliminate tumor cells bearing the target antigen (62). Blinatumomab (CD19xCD3) was the first BiTE to be licensed by the FDA for clinical use, initially in the treatment of acute lymphoblastic leukemia [reviewed in (63)]. The BiTe format was improved by addition of a disulphide bond to stabilize the variable domains of the two antigen-binding moieties; the resulting Dual-Affinity Re-Targeting protein (DART) has extended storage and serum stability (64). CD19xCD3 DARTs showed enhanced potency in directing B cell lysis over a single-chain format molecule with the same specificities, likely due to a higher association rate and target affinity (65).

HIV-specific BiTes and DARTs have recently been developed and tested *in vitro*. The BiTEs consisted of a scFV derived from either of the HIV gp120-specific bNAbs B12 and VRC01, the scFv of the antibody 17b, with or without two CD4 extracellular domains, CD4 (1+2); all were fused to an anti-human CD3ε scFV (66). The parent antibodies target conserved epitopes in Env. These constructs were tested for their capacity to suppress HIV replication in peripheral blood mononuclear cells or macrophages cultured with autologous CD8+ T cells. The B12 BiTe was the most potent, achieving >90% HIV inhibition at a concentration 10-fold lower than the other BiTes. Of note, the CD4(1+2) BiTE promoted infection of CD8+/CD4- T cells *in vitro*, possibly due to effects on gp120 conformation, whereas the other constructs did not.

Sung et al. reported the development of an HIVxCD3 DART comprising a non-neutralizing mAb against Env (A32 or 7B2) and a humanized anti-CD3 mAb. These HIVxCD3 DARTs were able to redirect *ex vivo* polyclonal CD8+ T cells from both HIV-seronegative and ART-suppressed HIV-seropositive donors to kill HIV-infected CD4+ T cells. Reduction in virus recovery even without the addition of CD8+ T cells (infected CD4+ T cells and DARTs only) was observed, which suggested that the DARTs could also recruit cytotoxic CD4+ T cells. The DARTs did not induce T cell activation or lysis in the absence of Env (67). Sloan et al. also developed a panel of HIVxCD3 DARTs based on the bNAbs PGT121 and PGT145, in addition to the nNAbs A32 and 7B2. These DARTs redirected CD8+ T cell killing of HIV-infected resting primary cells with low Env expression. Using *ex vivo* PBMCs from patients on suppressive ART a combination of two HIV DARTs was able to significantly reduce viral RNA in culture supernatants after 14 days in 3/4 subjects, suggesting that a subset of HIV reservoir cells may express enough Env to be targeted (68). In a third study, exposure of PBMC from ART-treated subjects to an HIV DART based on the VRC07 bNAb (VRC07-αCD3) was reported to upregulate cell surface Env. This was accompanied by a reduction in CD4+ T cells containing HIV DNA, suggesting that the DART both activated HIV reservoir cells and targeted them for cytolysis. In addition, short-term safety of a VRC07-rhesus CD3 DART was demonstrated in simian-human immunodeficiency virus-infected ART-treated macaques; importantly, no viral rebound was observed despite the possibility of viral reactivation by the DART (69). Based on pre-clinical work of Sung and Sloan, MacroGenics registered the first clinical trial of an HIV DART, MGD014, which will assess safety, tolerability and effects of the latent reservoir in ART-treated subjects (NCT03570918). A critical question is whether a combination of DARTs may be needed in order to achieve clinical benefit, given that viral escape driven by the selective pressure of anti-Env bNAbs was observed in a clinical trial in involving bNAb monotherapy followed by analytical ART interruption (70).

## Bispecific T Cell Receptors

A limitation of CAR T cells and bsAb therapies is their specificity for cell surface antigens. TCRs have the capacity to recognize intracellular proteins in the form of cell surface HLA class I-bound peptides. Soluble bispecific agents that combine T cell receptors (TCRs) with anti-CD3 effector function exploit both the vastly greater array range of potential targets (up to 9-fold more than antibodies) and the scalability of bsAb. Furthermore, picomolar affinity for the desired peptide-HLA (pHLA) complex can be achieved through targeted mutations in the complementarity-determining regions using phage display technology, generating molecules with higher potency than typical affinity-enhanced antibodies [(71), reviewed in (72)]. A stable soluble molecule is generated by truncation of the transmembrane domains and incorporation of a disulphide bond between the alpha and beta chains. The TCR is fused to an anti-CD3 scFV for redirection of T cells (as per BiTes and DARTS). Immune-mobilizing monoclonal TCRs Against Cancer antigens (ImmTAC) were shown to recruit non-cancer antigen-specific T cells to form an immunological synapse resulting in T cell activation and specific tumor cell lysis *in vitro* and *in vivo* (73, 74). The first ImmTAC to enter clinical trials is targeted to the melanoma antigen, gp100 and is in pivotal trials for the treatment of uveal melanoma (75) (NCT03070392).

The ImmTAC platform technology has been applied to HIV, specifically to the Gag protein, which is both highly conserved and abundant in infected cells, in contrast to Env. The first Immune-mobilizing monoclonal TCR Against Viruses (ImmTAV) was designed to target the HLA-A^*^0201-restricted Gag p17 epitope, SLYNTVATL (SL9) and known escape mutants (76). We reported that the HIV-specific ImmTAV molecule was able to redirect CD8+ T cells to kill Gag+ cells at nanomolar concentrations and low effector/target ratios. As with the bsAbs, the ImmTAV molecules were able to redirect CD8+ T cells from both HIV-seronegative and HIV-positive ART-suppressed donors to eliminate reservoir cells harboring inducible viruses (77).

The potential for ImmTAC and ImmTAV molecules to overcome low antigen expression was demonstrated by using tagged versions of the relevant TCRs to quantify pHLA complexes on cancer cells and virus-infected cells, respectively. These analyses showed that as few as 10 epitopes per cell could be quantified (77, 78). However, with such high TCR affinities the risk of off-target reactivity to self-peptide mimics must be carefully assessed in order to define a therapeutic window and avoid serious toxicity (79). Furthermore, conventional non-human toxicology testing is of limited predictive value, since TCR binding to peptide targets only occurs in the context of HLA class I molecules. This has been addressed by the development of comprehensive *in silico* and *ex vivo* human tissue analyses for pre-clinical safety evaluation (72). To date, ImmTAC molecules have been developed only for HLA-A02-restricted targets as this family of alleles is dominant in most ethnic groups across the world and is the best characterized. However, there is potential to extend the platform to other HLA alleles, which would broaden population coverage further.

## Future Directions

Over the past 5 years, the kick and kill concept has been tested in several clinical trials, with disappointing results. However, these studies have helped to refine our understanding of the dynamics and distribution of latent HIV reservoirs and have highlighted that each component of the strategy needs to be optimized. The licensing of CAR T cell and BiTe therapies is a reflection of astonishing progress in T cell-based immunotherapies for cancer in recent years. Forthcoming trials of HIV-specific versions of these agents will seek to assess whether clinically meaningful reductions in cellular reservoirs can be achieved with an “upgraded” kill. These trials could provide valuable insight into the levels of antigen required to sensitize cells to elimination by affinity-enhanced antibodies and TCRs, with and without latency reversal. The presence of archived CD8+ T cell escape mutants is perhaps the most obvious challenge for T cell retargeting therapy. Engineering specificity toward functionally constrained epitopes may mitigate against both past and future viral escape; however, we anticipate that combinations of agents will be needed, in an analogous fashion to combination ART. Furthermore, the efficiency of killing will depend on the functional capacity of non-HIV-specific T cells, which may be compromised in chronic HIV infection even after long-term ART (77, 80). This may require correction by immune checkpoint receptor blockade or other forms of immunomodulation.

Long-term follow-up of trial participants will be necessary to ascertain the requirement for repeated or continued administration of T cell retargeting therapy, a key consideration for its utility in clinical practice. Safety is also paramount since the risk/benefit ratio for people with well-controlled HIV is much higher than for individuals with cancer, who may have few treatment options. Early data suggested that gene-edited CD4+ cell infusions are well-tolerated. The risk of cytokine release syndrome (CRS) with CAR T cell administration in virologically suppressed patients is expected to be lower than that observed during treatment of malignancies, since Env is a non-self antigen and CRS is related to high (tumor) antigenic load. Furthermore, HIV BiTes and DARTs are based on monoclonal antibodies with an established safety profile. The exceptionally high affinity of ImmTAVs for pHLA will require cautious dose escalations in first-in-human trials. However, a major advantage of soluble agents over cellular therapies is that they are cleared within hours, enabling exposure *in vivo* to be tightly controlled. Human CD3 scFv has a short binding half-life (minutes) due to a nanomolar binding affinity. Experience with blinatumomab indicates this molecule does not cause off-target activation. On the other hand, on-target activation of CD4+ T cells by CD3 scFv could have the potential to spread infection, which highlights the need to protect uninfected cells by administration with effective ART, ideally including an integrase inhibitor. Lastly, retargeting therapies should be affordable and accessible to patients across the world. The soluble “single vial” bispecific agents have an advantage over cellular therapies with respect to cost and feasibility of manufacture at scale. A possible model for sustainable low-cost production is the recently announced strategic partnership between the International AIDS Vaccine Initiative and the Serum Institute of India, which aims to ensure global access to monoclonal antibody therapies (81).

## Author Contributions

HY, ZW, and LD contributed to the literature search and drafting of manuscript. ZW prepared the illustration.

### Conflict of Interest Statement

HY has received research funding from Immunocore Ltd. ZW and LD are employees of Immunocore Ltd.
